# Cancer of the vulva in Burkina Faso: a hospital-based case series

**DOI:** 10.1186/s13027-016-0080-y

**Published:** 2016-08-03

**Authors:** Nayi Zongo, Nina Korsaga-Somé, Amandine Banata Gang-Ny, Edgar Ouangré, Maurice Zida, Aimé Sosthène Ouédraogo, Aboubacar Hirrhum Bambara, Augustin Tozoula Bambara, Si Simon Traore, Pascal Niamba, Adama Traoré, Ahmadou Dem

**Affiliations:** 1 Division of General Surgery, Yalgado Ouédraogo University Hospital of Ouagadougou, 03 BP 7021 Ouagadougou, Burkina Faso; 2 Division of Dermatology and Venerology, Yalgado Ouédraogo University Hospital of Ouagadougou, 03 BP 7021 Ouagadougou, Burkina Faso; 3 Division of Pathologic Anatomy, Yalgado Ouédraogo University Hospital of Ouagadougou, 03 BP 7021 Ouagadougou, Burkina Faso; 4 Oncology Institute Joliot Curie of Dakar, Dakar, Senegal

**Keywords:** Cancer, Vulva, Demographics aspects, Diagnostic stages

## Abstract

**Background:**

Vulvar cancer is a rare gynaecological cancer. In Burkina Faso, the diagnosis of vulvar cancers is delayed and the prognosis is poor. However, no specific study on vulvar cancers has been conducted at the moment. This work aimed to study the characteristics of these cancers.

**Methods:**

This is a prospective study on histologically confirmed primary cancers of the vulva diagnosed between 1st January 2013 and 30th June 2015. The demographic and clinical aspects were studied at the Yalgado Ouedraogo University Hospital of Ouagadougou (CHU-YO).

**Results:**

We noticed 21 cases of vulvar cancers within 30 months, ranking it as the 4th most common gynaecological cancer. The average age of the patients was 55 years (standard deviation +/− 6.3) and the median age was 57 years. Scars resulting from female circumcision, menopause (*n* = 20) and HIV infection were noticed in 19 cases and 6 cases respectively. The average time from first symptoms to first consultation was 29 months. Pain and ulceration were the main reasons for consultation. The clinical picture was chiefly an ulcero-granulating tumour. There was squamous cell carcinoma in 20 cases and basal carcinoma in 1 case. Fifteen patients were at stage III or IV, where of three patients had metastatic disease. We noticed vitiligo in 9 vulvar cancer cases.

**Conclusion:**

The cancer of the vulva is rare. Women are of menopausal age, are mostly circumcised and HIV-infection is common. A majority of patients sought consultation at advanced stage of disease, and diagnosis was belatedly made. Pain and ulceration were the main reasons for consultation. The sensitization of the population, education for self- examination would allow earlier diagnosis.

## Background

Vulvar cancer is a rare pathology representing 3 to 5 % of gynaecological cancers [1, 2]. It mainly affects menopausal and oestrogen-deficient women [2]. The average age of its occurrence is 60 years [3, 4]. Cases were found in young women [5, 6]. Buttmann-Schweiger and Baantrup noticed 11.5 and 23 % of vulvar cancers respectively in women below 50 [5, 7]. Still, its incidence is increasing strongest in women aged <60 years [6, 8]. Two aetiological forms are classically described: the dystrophic vulvar cancer in elderly women (sclerotic lichen) and the cancer developed on a healthy vulva due to human papilloma virus infection (HPV) in younger women [9, 10]. Main other risk factors are HIV infection, and tobacco addiction [11]. Squamous cell carcinoma is the main histological type [12]. The diagnostic stages are different between Europe and Africa with 47.6 % for stage IB in the Netherlands [13] and 45.4 % for stage III in Nigeria [14]. In Burkina Faso, no specific study on vulvar cancers has been conducted at the moment. However, cervical cancer and HPV-infections, one of the main risk factors of cervical and vulvar cancers are frequent. In addition invasive lesions are not appropriately managed, because of poverty. The frequency of HPV lower genital tract infection is 42.1 % in women under 30 and 57.9 % in women over 30 in Ouagadougou (PCR multiplex) [15]. In Western Africa, other studies reported HPV prevalence for women with normal cytology. The highest prevalence of HPV (any type) was found in Guinea (47.9 %) using PCR technology [16].

In this context which characterizes developing countries, this work is being carried out to specify demographic and clinical characteristics of this cancer at the Yalgado Ouédraogo University Hospital of Ouagadougou (CHU-YO).

## Methods

### Type of study

This was a case-series-study, based on primary cancers of the vulva at the Yalgado Ouédraogo University Hospital of Ouagadougou (CHU-YO). It was carried out over the period from 1st January 2013 to 30th June 2015.

### Site of the study

The study took place in Burkina Faso, a country with limited resources, located in the heart of West Africa. The data were collected in the departments of General Surgery, Dermatology-Venereology, and Gynaecology-Obstetrics of Yalgado Ouédraogo University Hospital of Ouagadougou (CHU-YO). This Hospital is the most representative health care reference center in Burkina Faso. It is the national reference hospital for the diagnosis and management of cancers as well. The three above-mentioned departments together with the laboratory of anatomy-pathology are the 4 departments involved in managing cases of vulvar cancers at Yalgado Ouédraogo University Hospital.

### Population of the study

All the patients who were hospitalized at CHU-YO for vulvar lesions were concerned.

### Criteria of inclusion

We included in this study all the patients who were carriers of histologically confirmed primary vulvar cancers. Were excluded cases of secondary vulvar cancers.

### Procedures of data collection

The data collection was carried out by giving out a questionnaire to the patients who were all examined by an oncologist surgeon, a dermatologist and/or a gynaecologist. From each patient, sociodemographic data were collected (age, sex, place of residence), vulvar cancer risk factors (history of genital infections like genital candidiasis, trichomoniasis and chlamydia, HIV infection, menopause, tobacco addiction, diabetes, arterial hypertension, atherosclerosis, sclerotic lichen, pemphigus vulgaris, lupus, history of personal or family cancers, scar of female circumcision). The time from first symptoms to first consultation and the circumstances of admission were also sought. The physical examination revealed an elementary lesion and the location and presence of adenopathy. The biopsy and histological examination helped confirm the diagnosis. The evaluation of the extension was made thanks to a pulmonary radiography and anabdominopelvic echography, and even better when necessary, a thoraco-abdominopelvic tomodensitometry examination was performed.

### Analysis of the data

The cancers were classified in accordance with the 2010 WHO TNM and the classification of the International Federation of Gynaecology and Obstetrics (FIGO).

## Results

In 30 months, 2187 cases of cancers were registered in women. During the same period, 396 cases of gynaecological cancers were diagnosed. 21 cases involved primary cancers of the vulva, i.e., 5.3 % of all the gynaecological cancers. The cancer of the vulva was considered as the 4th most common diagnosed gynaecological cancer just after that of the cervix (222 cases), the uterus (79 cases), and the ovary (74 cases).

The patients’ average age was 55, with range 32–60 years (standard deviation +/− 6.3) (Table 1). The median age was 57 years. Nineteen patients were circumcised just from their childhood and were having the scars. All the wounds resulting from this female circumcision were traditionally treated by the repeated application of a strong heat (hot water and /or heated stone). Six patients were HIV-1 positive, one patient had a coinfection of HIV-1 and HIV-2 (Table 2). The average time from first symptoms to first medical consultation was 29 months, ranging from 12 to 60 months (Table 1).

**Table 1:** Clinical and histopathological characteristics *n* = 21

	Categories		Number
Age (year)	➢ 50		20
	<50		1
Median age	57		-
Average age	55		-
Time from first symptoms to first medical consultation (month)	12–24		9
	24–36		4
	36–48		5
	48–60		3
Reasons for consultation	inability to sit down		2
	perineal pain		9
	smelly perineal oozing		2
	inguinal swelling		5
	ulceration		9
	painful urination		1
	rectal bleeding		2
Histological type	squamous cell carcinoma		20
	basal cell carcinoma		1
Stages	TNM	FIGO	
	T1bN0M0	Ib	1
	T2N0M0	II	2
	T2N2bM0	IIIb	4
	T2N2bMx	IIIb	5
	T3N3Mx	IIIc	2
	T2N2bM1	IVb	4
	T2N2aM0	IIIa	3

*TNM* tumor-nodes-metastasis, *FIGO* international federation of gynecology and obstetrics

**Table 2:** Distribution of patients according to risk factors, *n* = 21

Risk factors	Number
HIV	6
History of genital infection	21
Menopause	20
Diabetes	2
High blood pressure	3
Multiple sexual partners (polygamy)	15
Early sex (<19 years)	21
Female circumcision scar	19
Squamous cell carcinoma (front)	1

**Fig. 1:**
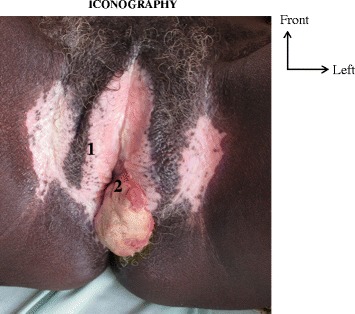
1 - vitiligo. 2 - tumor

**Fig. 2:**
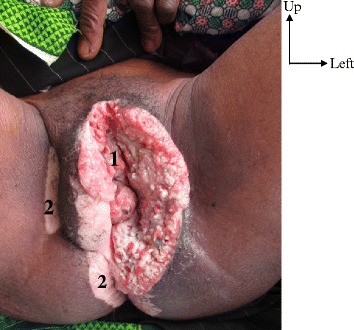
1- tumor. 2- vitiligo

**Fig. 3:**
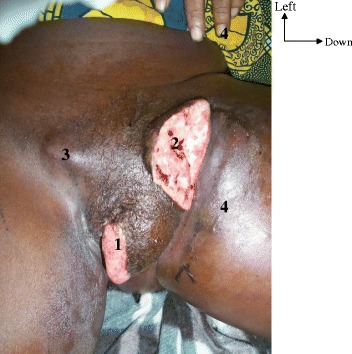
1-ulcero-granulating tumor. 2-fixed right inguinal adenopathies. 3-ulcerated left inguinal adenopathies. 4-lymphoedema

Pain and ulceration were the main reasons for consultation (Table 1). A pronounced symptomatology from loco-regional expansion of the tumor was the reason for consultation in 4 cases: There was dysuria (1 case), impossibility of sitting down (2 cases), and putrid discharge (2 cases). The clinical aspect was an ulcero-granulating tumour (14 cases), an ulcerated tumour (3 cases) and an ulcero-necrotic tumour (4 cases) (Figs. 1 and 2). The tumour was coated with a whitish matter in 2 cases. The localization was clitoral in one case, minor labial in 2 cases, major labial in 8 cases and mixed labial in 5 cases. The tumour occupied the whole vulva in 5 cases. The anus was clinically invaded in 3 cases and there was urethral meatus in 2 cases. A vitiligo located at the level of the labia and/or of the root of the thighs was present in 9 cases (Figs. 1 and 2). Inguinal adenopathies were present in 16 cases. They were fixed in 7 cases and fistulated in 3 cases (Fig. 3).

Squamous cell carcinoma was involved in 20 cases and basocellular carcinoma in 1 case. Three of the patients had metastases and in 7 cases, the evaluation of the extension could not be done. From all 21 patients, 18 women were presented at stages III and IV. Stage I and stage II of FIGO were noticed in 1 and 2 cases, respectively. The diagnostic stages are summarized in Table 1.

## Discussion

In 30 months, we collected 21 cases of primary cancers of the vulva at CHU-YO. These cancers are actually rare. They represented 5.3 % of all the gynaecological cancers diagnosed in the same period (396 cases). In Africa, all the authors agree that this cancer is rare but its proportion in relation to gynaecological cancers is highly variable, oscillating between 1.3 and 5 % [14, 17, 18]. In our study, cancer of the vulva was the 4th most common diagnosed gynaecological cancer in order of importance after cancer of the cervix, the uterus, and the ovary. In the literature, it is either the 4th or 5th gynaecological cancer [11, 19, 20]. The cancer of the uterus ranks first in the west European countries [21]. In Africa, it is that of the cervix [11, 20, 21]. The results from our study are based clinical observations, and might not be directly comparable with the ranking of incident cancers from cancer registries. The type and frequency of the factors favoring this situation vary from one country to another.

The cancer of the vulva is, classically speaking, a disease of elderly and menopausal women [1]. The average age of our patients was 55. In Africa, the average age varies between 46 and 61 [19, 22, 23]. Some average ages of 46 and 49 were noticed in Ghana and in Mali [19, 22]. But, in the western European, the average ages are over 70 [5] and in Overall, 55.7 % of the women were diagnosed at the age of 70 years and above [6]. The difference of the average ages between Europe and the less developed countries could be explained, by the difference of life expectancy at birth, which is 59 years in Burkina Faso against 85 years in France [24, 25] but also by the frequency of HPV infection in Africa [15, 16].

Two aetiological forms of vulvar cancer are classically described:

- The cancer of menopausal and elderly women resulting from chronic genital inflammatory diseases, associated with a mutation of gene P53. This cancer is frequent in developed countries where life expectancy is high [5, 6];
- The cancer developed from intra epithelial neoplasia due to the human papillomavirus infection [9, 10]. Each of our patients had already at least an episode of genital infection.

HIV infection was noticed in 6 out of 21 patients. The youngest of whom was 32. This rate is very high compared to the average prevalence rate of HIV infection in the adult population of Burkina Faso which is 1 % [26].

The HIV infection, through the resulting immunodepression is suspected to predispose to the cancer of the vulva [10]. The correlation between neoplasia of the low genital tractus and HIV is so important that the Center for Disease Control and Prevention has included high-grade dysplasias and in situ carcinomas as well, in the classification of HIV, with the invasive cancer of the cervix considered as a condition defining AIDS. Intra epithelial neoplasia of the vulva is also more frequent in HIV-infected women with a relative risk of 29 compared to the group of HIV-uninfected women [27]. Although human papillomavirus is strongly suspected to play an etiologic role in younger women [9, 28, 29], prevalence could not be confirmed in our series, because of the high cost of the diagnostic test. However, a previous study in our country noticed a strong relation between HPV infection and invasive cancers of the cervix [15].

HPV is one of the main risk factor of carcinomas of the low genital tractus. This led to some hope in the vaccines directed against the most frequent genotypes in Europe (16 and 18) [30]. The newly developed 9-valent vaccine [31] might be promising in the actual working context, because HPV genotypes seem to vary from one country to another, with the predominance of genotypes 52 (29.4 %) and 58 (20.6 %) in Ouagadougou [15]. A part from the infectious factors, many other risk factors were reported in the literature. Diabetes, obesity, hypertension, and atherosclerosis, sclerotic lichen, lupus, pemphigus vugaris are suspected to be the factors which are most frequently associated with vulvar cancers [11, 23]. In addition to these factors, in the researcher’s series, other factors that could be involved in the appearance of vulvar cancers were noticed. Nineteen (19/21) of the patients were having a scar due to female circumcision. The prevalence of female circumcision in the general population of those over 50 is not known in Burkina Faso. However, it is of 76 % among the age group 15–49 years and of 13.3 % in those below 15 [32]. Traditionally, the scars due to burns are risk factors of cutaneous carcinoma (Marjolin’s tumour) [33]. Yet, it has still not been reported in the literature that female circumcision can develop vulvar cancers. But the traditional use of heat repeatedly in order to heal wounds due to female circumcision could occasion real burns. Thus, the scars resulting from female circumcision could actually be like real scars due to burns, risk factors of carcinoma. However, from our observational study we cannot distinguish whether these are independent risk factors or merely coexisting medical conditions common to women of this age.

Similarly, the researcher could not come across, in the literature, cases of cancers of the vulva grown on lesions of vitiligo. However, this coexistence in almost half of the patients (9 cases/21), makes the researcher wonder whether vitiligo is not a risk factor of vulva cancer or whether both pathologies were not supported by the same subjacent physiological condition. Besides, though the relation with vitiligo is not well defined in the literature, some authors noticed this association. As it is reported in Carii et al., among the patients who presented an association of sclerotic lichen and vulvar cancer, 16 % had an antecedent of vitiligo [34].

From a clinical point of view, a mass, a plaque, an ulcer, a nodule, a genital pruritus, bleedings and post-menopausal vaginal losses, dyspareunia and inguinal adenopathies can be occasions for discovery [12]. Pain, ulceration, and tumefaction were the main reasons for consultation by our patients.

In the literature, the main localization remains the major labia. In 10 % of the cases, the lesions are extremely extensive and the primary site is difficult to define [9] as it is in 5/21 cases of our patients.

Just as it is in our series, the clinical aspect is chiefly an ulcero-granulating tumour in the literature [35]. All the authors agree on the most frequent histological type which is asquamous cell carcinoma [2, 34]. This is the case in our study in which there were 20 squamous cell carcinomas against only one basocellular carcinoma.

The evolution of vulvar cancers has long been locoregional, the ganglionary metastasias are premature and the distant metastasias are late [36]. In our study, only two patients (2/21) were at stage I or II contrasting with a small proportion of metastatic cases (3/21). These results are similar to the data in the African literature in which the time to first medical consultation was superior to 6 months in 60 % of the cases and 80 % of the patients were at stages III and IV of FIGO [14, 19, 37]. This contrasts with stage at diagnosis in the developed countries. In the USA, 90 % of cases are diagnosed in situ or at a premature stage of the invasive disease [38]. The diagnoses which are late, in the African environment might be due to the lack of awareness-raising [11] and mostly the sociocultural context. Indeed, in our context, the vulva is considered an area of decency and the patients only come for consultation when there appears an unbearable sign. This is the case of two of our patients who came on consultation when they saw a putrid perineal discharge and when they could not sit down. Unfortunately, when these signs appear, the tumor is already at an advanced stage. Let us also note the responsibility of some health workers who might diagnose the disease on time, just doing local care. Although there is no systematic screening for the cancer of the vulva, patients with history of cervical disease, might benefit from regular watch over. Likewise, patients with sclerotic lichens or with history of intraepithelial neoplasia should be educated to examine themselves through a mirror [2].

## Conclusion

The cancer of the vulva is rare. Women are of menopausal age, are mostly circumcised and HIV-infection is common. A majority of patients sought consultation at advanced stage of disease, and diagnosis was belatedly made.

Pain and ulceration were the main reasons for consultation. It was essentially an ulcero-granulating tumour. The sensitization of the population, education for self- examination, the treatment of precancerous lesions and watching over the patients at risk (HIV infection) would allow earlier diagnosis. Vaccination remains the best way of prevention, though is it too expensive for a less-developed country like Burkina Faso.

## Abbreviations

AIDS, acquired immune deficiency syndrome; FIGO, International federation of gynecology and obstetrics; HIV, human inmmunodeficiency virus; HPV, human papilloma virus; TNM, tumor nodes metastasis.
